# Digital Phenotyping to Enhance Substance Use Treatment During the COVID-19 Pandemic

**DOI:** 10.2196/21814

**Published:** 2020-10-26

**Authors:** Michael Hsu, David K Ahern, Joji Suzuki

**Affiliations:** 1 Department of Psychiatry Brigham and Women's Hospital Boston, MA United States; 2 Digital Behavioral Health and Informatics Research Program Brigham and Women's Hospital Boston, MA United States; 3 Division of Addiction Psychiatry Brigham and Women's Hospital Boston, MA United States

**Keywords:** digital phenotyping, digital psychiatry, addiction, psychiatry, coronavirus, COVID-19, digital health, treatment, drugs, substance use disorder

## Abstract

Due to the COVID-19 pandemic, many clinical addiction treatment programs have been required to transition to telephonic or virtual visits. Novel solutions are needed to enhance substance use treatment during a time when many patients are disconnected from clinical care and social support. Digital phenotyping, which leverages the unique functionality of smartphone sensors (GPS, social behavior, and typing patterns), can buttress clinical treatment in a remote, scalable fashion. Specifically, digital phenotyping has the potential to improve relapse prediction and intervention, relapse detection, and overdose intervention. Digital phenotyping may enhance relapse prediction through coupling machine learning algorithms with the enormous amount of collected behavioral data. Activity-based analysis in real time can potentially be used to prevent relapse by warning substance users when they approach locational triggers such as bars or liquor stores. Wearable devices detect when a person has relapsed to substances through measuring physiological changes such as electrodermal activity and locomotion. Despite the initial promise of this approach, privacy, security, and barriers to access are important issues to address.

## Introduction

The COVID-19 pandemic has presented unprecedented challenges to the addiction community. First, drug-seeking behaviors can increase exposure to COVID-19. The converse is also true in that pulmonary sequelae from methamphetamine or tetrahydrocannabinol (THC) use may confer higher risk for contracting COVID-19 and may lead to more severe comorbidities for those infected with the virus [1]. Secondly, the COVID-19 pandemic is likely heightening the risk of substance use relapse [2]. Many substance users now live in greater isolation with fewer social supports, diminished access to substance use treatment, and fewer distractions from substance use [3]. Quarantine and social distancing are associated with fear, anxiety, and boredom, which are known risk factors for relapse [4]. Finally, the COVID-19 pandemic has limited our ability to monitor patients’ progress and deliver adequate care. In our outpatient addiction center, we have suspended toxicology testing, which was critical in monitoring adherence to treatments such as buprenorphine. Whereas we previously relied heavily on in-person group treatment, most visits are now conducted on the internet and on an individual basis.

To address the clinical gap engendered by the pandemic, we recommended that our patients pursue web- or audio-based Alcoholics Anonymous and Narcotics Anonymous groups, which are largely unvetted substitutes, as well as evidence-supported web-based therapies such as cognitive behavioral therapy [5,6]. The Substance Abuse and Mental Health Services Administration (SAMHSA) and Drug Enforcement Administration (DEA) have administered policies enabling virtually supported, take-home buprenorphine induction and have created opportunities for patients to access a buprenorphine hotline [7] and virtual bridge clinic (currently implemented at our institution). Whether these efforts can serve as adequate substitutes for in-person addiction treatment remains to be seen; meanwhile, we need additional, scalable strategies to assist in substance use monitoring and treatment in ways that are practical and acceptable.

**Figure 1:**
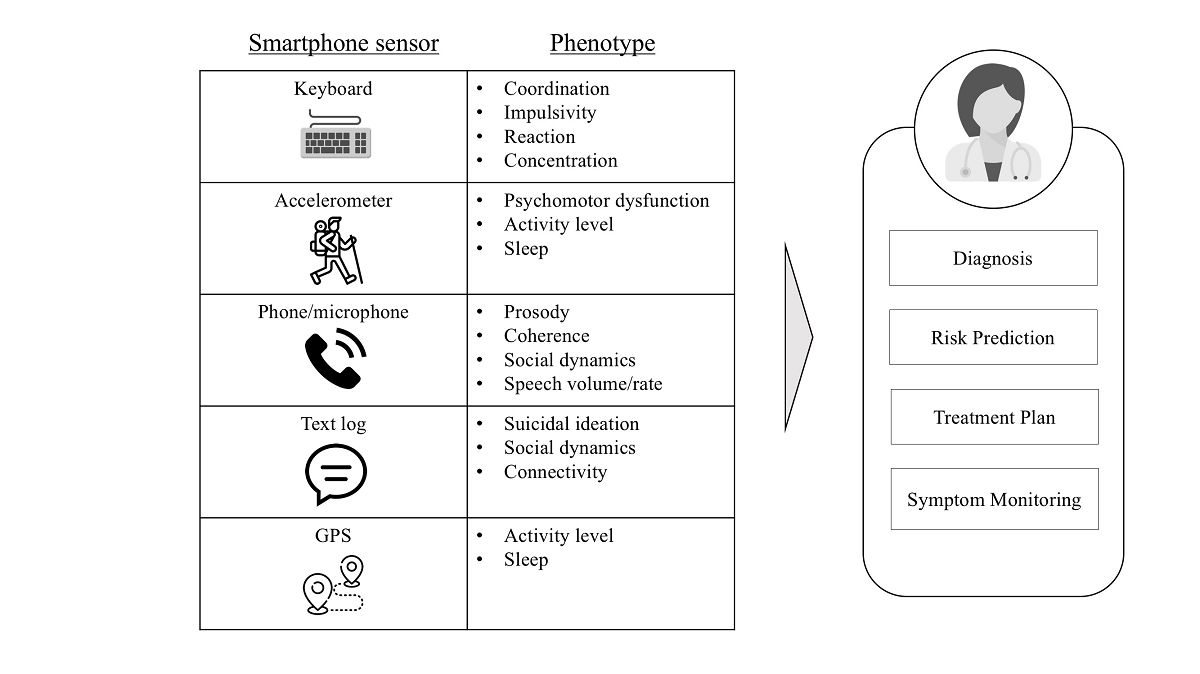
Digital phenotyping implements smartphone sensors to collect passive data that can then inform clinical diagnosis, risk prediction, treatment, and symptom monitoring [11].

Digital phenotyping or behavioral sensing [8] uses passively collected, real-time data (eg, GPS tracking, social patterns, typing patterns) from patients’ smartphones to inform clinical assessment, predict changes in clinical status, and deliver on-demand interventions in a scalable, cost-effective manner (Figure 1). Smartphone ownership is nearly ubiquitous in the United States, even among individuals with substance use disorders, and possesses a vast array of functionality that can be leveraged for clinical purposes [9,10].

Digital phenotyping is an especially attractive clinical tool for substance use treatment during the COVID-19 pandemic, as it not only operates remotely but is also convenient for patients (no active input required), is not administratively burdensome, and may be an effective way to address historic challenges in detecting problematic substance use behaviors and delivering timely clinical interventions. Digital phenotyping has shown initial success in relapse prediction for schizophrenia [12], bipolar disorder [13,14], and mood disorder detection [14]. We describe three broad applications of digital phenotyping for addiction treatment, highlighting their potential clinical use, state of evidence, and next steps for implementation.

## Application 1: Relapse Prediction and Intervention

Digital phenotyping has significant potential to enhance relapse prediction. Even before the COVID-19 pandemic, relapse was common, with prevalence rates ranging from 40% to 75% depending on the substance and the definition used in the study [15-17]. Machine learning tools based on survey responses of demographics, alcohol use, and psychological factors have 77% predictive accuracy for alcohol relapse [18]. Activity-based analysis, also known as location-based activity, analyzes how individuals’ locational or GPS data correlate with their affect, thoughts, and behavior. Dr David Epstein’s group combined passive geographical input with machine learning tools to predict opioid drug craving or stress 90 minutes into the future among patients with opioid use disorder on maintenance buprenorphine or methadone; they achieved a positive predictive value of 0.93 [19]. Another ongoing study by Curtin et al [20] implements digital phenotyping based on machine learning tools and contextualized static and dynamic risk signals to predict lapse in opioid use disorder. Activity-based analysis can theoretically be personalized based on regions of risk that are specific to the individual.

Automated messages can be sent to patients who are flagged as being at high risk of relapse, including warning messages, motivational messages, or recommendations to schedule follow-up appointments. A-CHESS (Addiction-Comprehensive Health Enhancement Support System), a multifeature randomized controlled trial–backed mobile app suite that supports alcohol recovery, delivers an alert to patients when their GPS indicates they are approaching a liquor store or bar [21]. Clinicians can also use this data to triage limited administrative resources by prioritizing follow-up to higher-risk patients.

While digital phenotyping shows initial promise in enhancing relapse prediction, nearly all studies to date have been pilot studies conducted in selected patient populations. Further studies highlighting population-level outcomes such as hospitalization and prediction accuracy, especially when coupled with clinical interventions, are needed. Prior to clinical implementation, patient acceptability and feasibility of use in the clinical setting should be further explored. Other dynamic factors for substance use relapse, such as one’s social environment, can be incorporated to fine-tune the predictive power of these interventions [22].

## Application 2: Relapse Detection

Digital phenotyping can potentially be used to detect when a patient has relapsed. One pilot study of 30 emergency room patients demonstrated that a biosensor that collects electrodermal activity, locomotion data, and skin temperature was able to detect opioid use and distinguish between heavy and nonheavy opioid users [23]. Another study describes a wearable biosensor that can monitor alcohol consumption through detection of ethyl glucuronide in human sweat, although further population-based studies are needed to establish its acceptability and efficacy [24]. Heart-rate variability (HRV) has also been shown to be linked to alcohol use and smoking; however, specificity is an issue, as HRV is also impacted by affective disorders and trauma [25].

Once the system has detected potential relapse, an automated message can then be relayed to accountability partners (eg, family or friends), front-line staff, or clinicians. Third parties can reach out to patients who have a strong signal of relapse or repeated signals of relapse to confirm and ascertain the nature of the relapse, provide counsel over the phone, set up follow-up appointments, or offer appropriate addiction services. Wearable biosensors could potentially be used as a proxy for urine toxicology screening when monitoring for substance use, especially for patients at high risk for contracting COVID-19. Importantly, clinicians should empower patients to decide how their data will be used and obtain appropriate consent before it can be shared with third parties.

## Application 3: Early Overdose Detection

Physical distancing during the COVID-19 pandemic can be life-threatening for individuals who use opioids due to increased overdose deaths from using in isolation, supply chain disruptions, and increased relapse [7,26]. Nandakumar et al [27] developed a potentially lifesaving contactless mobile phone app to detect opioid overdose by measuring respiratory rate using a short-range active sonar through a mobile phone. Their algorithm was able to detect 19/20 (95%) of simulated overdoses in the operating room. Although this proof-of-concept app is promising, further population-based studies are needed to optimize and demonstrate its efficacy.

In theory, a device could automatically alert emergency medical services (EMS), authorized friends, and clinicians of a potential opioid overdose. Providers should empower patients to decide who is notified of the overdose, as patients may not want EMS or police to be automatically informed. Instead, they may prefer friends, neighbors, providers, or other public health professionals to be alerted first. In this case, third parties can check in with the patient, and if there is no response within a certain time frame and appropriate concern for overdose, they can administer naloxone (if they are trained to do so and are in the vicinity) and consider whether to bring the patient to the emergency room or involve first responders.

## Limitations to Digital Phenotyping

There are several notable limitations to digital phenotyping and barriers to implementation that should be addressed. First, security and privacy are critically important concerns. Data stored on a mobile phone or cloud service may be vulnerable to security threats such as password compromise, while data uploaded to an electronic medical record system may be accessed by third parties [11,28]. Although call log, GPS, and accelerometer data are often anonymized by application developers, a theoretical risk remains that third parties can identify individuals based on raw data [29]. Clinicians should thoroughly vet digital phenotyping applications based on the App Evaluation Model developed by the American Psychiatric Association to ensure the safety and privacy of identifiable patient data [30,31]. Providers should protect patient data and counsel patients regarding privacy risks, especially for vulnerable individuals such as those with mental illness or substance use disorders.

Second, further studies are needed to assess the acceptability and feasibility of digital phenotyping among patients with substance use disorders. As is the case with all digital health technologies, the clinical efficacy of an app does not always translate to user adoption. Lowering barriers to entry such as improving user friendliness and addressing technology illiteracy are important.

Third, inequality of access to smartphone technology is problematic, especially among individuals who are of lower socioeconomic status, aged 85 years or older, widowed, Medicaid recipients, Black or Hispanic, and have disabilities [32]. However, many state Medicaid programs now offer free mobile phones to eligible recipients, and federal programs such as the Federal Communications Commission’s Lifeline Program offer a subsidy for cellular and data service plans to low-income individuals [33,34]. Lack of broadband access may be a barrier, especially for rural and underserved communities [35]. Further research on these barriers to access is needed to devise apt solutions to equip even the most marginalized and vulnerable populations with digital technology.

Finally, there are salient administrative and financial barriers to clinical implementation. While there may be long-term cost savings in the reduction of health care use and less labor-intensive means of substance use monitoring, introducing any new health technology requires an initial resource investment. Patients and staff need to be trained on the technology and the clinical integration of the data streams generated by digital phenotyping applications. To move beyond resource-rich academic and research settings, clinician time and use of digital phenotyping will need reimbursement by payors, which requires demonstrating the clinical and financial value of the technology to insurance companies. This supports the call for methodologically rigorous studies to determine how the initially promising experimental outcomes of digital phenotyping translate to real-world clinical value.

## Conclusions

Digital phenotyping has tremendous potential to augment substance use treatment, especially during the COVID-19 pandemic. Specifically, this technology can help address significant challenges in improving care for substance use disorders in the areas of relapse prediction and intervention, relapse detection, and overdose detection. Despite the promise and potential of digital phenotyping, many studies thus far have been proof-of-concept or pilot studies in siloed patient populations; more robust, generalizable experiments are needed to demonstrate clinical efficacy, acceptability, and feasibility. Iterative observation and experimentation can allow for further refinement of the underlying technology and how it will be clinically integrated. Concerns of privacy, security, equity, and sustainability need to be addressed.

## Abbreviations

A-CHESS: Addiction-Comprehensive Health Enhancement Support System
DEA: Drug Enforcement Administration
EMS: emergency medical services
HRV: heart-rate variability
SAMHSA: Substance Abuse and Mental Health Services Administration
THC: tetrahydrocannabinol
